# Targeted Polymeric Micelles System, Designed to Carry a Combined Cargo of L-Asparaginase and Doxorubicin, Shows Vast Improvement in Cytotoxic Efficacy

**DOI:** 10.3390/polym16152132

**Published:** 2024-07-26

**Authors:** Igor D. Zlotnikov, Elena V. Kudryashova

**Affiliations:** Faculty of Chemistry, Lomonosov Moscow State University, Leninskie Gory, 1/3, 119991 Moscow, Russia; zlotnikovid@my.msu.ru

**Keywords:** combined formulation, L-asparaginase, doxorubicin, Aikido principle, synergy, catalytic activity

## Abstract

L-asparaginases (ASP) and Doxorubicin (Dox) are both used in the treatment of leukemia, including in combination. We have attempted to investigate if their combination within the same targeted delivery vehicle can make such therapy more efficacious. We assembled a micellar system, where the inner hydrophobic core was loaded with Dox, while ASP would absorb at the surface due to electrostatic interactions. To make such absorption stronger, we conjugated the ASP with oligoamines, such as spermine, and the lipid components of the micelle—lipoic and oleic acids—with heparin. When loaded with Dox alone, the system yielded about a 10-fold improvement in cytotoxicity, as compared to free Dox. ASP alone showed about a 2.5-fold increase in cytotoxicity, so, assuming additivity of the effect, one could expect a 25-fold improvement when the two agents are applied in combination. But in reality, a combination of ASP + Dox loaded into the delivery system produced a synergy, with a whopping 50× improvement vs. free individual component. Pharmacokinetic studies have shown prolonged circulation of micellar formulations in the bloodstream as well as an increase in the effective concentration of Dox in micellar form and a reduction in Dox accumulation to the liver and heart (which reduces hepatotoxicity and cardiotoxicity). For the same reason, Dox’s liposomal formulation has been in use in the treatment of multiple types of cancer, almost replacing the free drug. We believe that an opportunity to deliver a combination of two types of drugs to the same target cell may represent a further step towards improvement in the risk–benefit ratio in cancer treatment.

## 1. Introduction

The therapeutic use of antitumor enzyme preparations that catalyze the depletion of certain essential amino acids is based on data on the absence or low activity of the corresponding amino acid synthases in tumor cells [1,2,3]. L-asparaginases from *E. coli* (EcA) and *Erwinia chrysanthemi* (ErA) are used in the first line of standard therapy for acute lymphoblastic leukemia, as well as in the treatment of other types of tumors [4,5,6,7,8]. The main mechanism of L-asparaginase antitumor activity is its ability to inhibit protein and nucleic acid synthesis by hydrolyzing asparagine in plasma, resulting in the formation of aspartic acid and ammonia [9]. Lymphoblast cells, which lack asparagine synthase and are therefore unable to synthesize L-asparagine independently, like healthy cells, have been shown to be particularly sensitive to L-asparaginase treatment [10]. Additionally, it has been found that L-asparaginase-mediated antitumor activity extends beyond simply reducing the concentration of free L-asparagine. There are also other more specific mechanisms at play, such as the uptake of L-asparaginase into cancer cells and the inhibition of telomerase activity [11,12].

However, the long-term therapeutic use of protein preparations, especially those derived from bacterial sources, causes the development of immunological hypersensitivity, including anaphylaxis. L-asparaginases can be hepatotoxic and neurotoxic [6,7,13,14].This can cause therapy to need to be discontinued prematurely [15,16,17]. Additionally, enzyme preparations often have limited effectiveness and are therefore used in combination with other drugs, such as Doxorubicin (Dox), for optimal results [8,18,19,20]. Increasing the tolerability of ASP drugs by increasing their activity, as well as reducing immunogenicity, could contribute to a more complete realization of their therapeutic potential.

The stealth effect is significant in terms of enhancing the pharmacokinetic characteristics of a drug formulation, improving targeted delivery, and reducing toxicity due to its practical invisibility to immune cells. Initially, the term was used to describe the behavior of pegylated liposomes, but it has been applied to a wider range of drug delivery systems [21,22,23,24]. To extend the duration of a drug’s activity and maintain its concentration within the therapeutic range, it may be beneficial to utilize stealth technologies [25]. In this study, the stealth effect was achieved for ASP through the shielding of immunogenic epitopes using cationic polymers and inclusion in the micellar system, which also shields the enzyme epitopes. This modification has the potential to improve the properties of ASP and establish it as a leading drug in its class.

Cationic [26,27,28] and anionic [29,30,31] polymer particles are actively used for drug delivery, and in addition, the use of zwitterionic [32] polymers is actively developing). Zwitterionic formulations enable the combination of the benefits of each component to achieve maximum therapeutic efficacy. For example, a zwitterionic polymer comprising monomers such as carboxybetaine methacrylate, methacrylohydrazide, and sodium methacrylate (SMA) that were polymerized using AIBN (azobisisobutyronitrile) was applied. In addition, Dox (doxorubicin) was encapsulated within the resulting polymer to create a micellar structure. The positive charge of Dox was balanced by the negative charge of the polymer, resulting in a more stable and effective nanocarrier system. In vivo studies showed that the drug carrier effectively reduced tumors by up to 55%, without causing significant toxicity [30]. In addition to zwitterionic polymers, multilayer polymer particles are popular carriers: for example, chitosan-hydroxypropyl methylcellulose or chitosan-alginate multilayer particles. Such systems are characterized by increased stability and often have stimulus (including pH) sensitivity

In this study, we applied another approach where a cationic-modified enzyme interacts with anionic polymeric micelles. The systems we present are superior to those based on ionic polymers, as we can vary the charge of the Dox containing micelles and ASP, thereby controlling solubility and binding affinity for the drug being loaded, regulating the binding of the enzyme depending on the enzyme surface charge, and thereby modulating the pH optimum of catalytic activity. Additionally, the proposed systems, based on heparin micelles, have the potential to combat thrombosis, which is relevant in the context of leukemia.

Recently, we have developed a novel type of “smart Aikido micelle” that targets cancer cells due to their unique properties in the tumor microenvironment. This is achieved through the selective release of Dox, which is triggered by the breaking of the S-S bond mainly within cancer cells. These smart micelles exhibit remarkable properties, such as increased cancer cells permeability, pronounced enhanced cytotoxic effects of Dox, and high selectivity index towards cancer cells in relation to healthy cells. Currently, there are RedOx-responsive polymeric micelles that can respond to internal RedOx potential in order to ensure targeted drug delivery and controlled release of drugs at the site of the tumor [29,33,34,35,36]. In addition, redox sensitivity, specifically the sensitivity of polymeric nanoparticles to glutathione (GSH), ensures the presence of labile disulfide bonds between polymer chains or between the polymer and the drug. Glutathione is the most significant antioxidants in cells. It is present in all cellular compartments at millimolar concentrations (1–10 mM) and plays a dual role in cancer, both protecting and contributing to pathogenesis. Glutathione participates in the detoxification of carcinogenic substances, and alterations in this process can significantly affect cell viability. Increased concentrations of glutathione accumulate in cancer cells and can lead to resistance to antitumor agents (cytotoxics) [37,38,39]. “Smart” micelles exploit this characteristic of cancer cells; glutathione serves as a trigger for accelerated release of the cytotoxic agent. Due to the use of RedOx-sensitive micelles, a high selectivity of cytotoxic formation to tumors is achieved in comparison with normal cells.

Inspired by these findings, we have decided to further promote this approach through its application to enhance the therapeutic potential of asparaginase drugs (ASP), which are used in the treatment of leukemia. Although these drugs are used in standard leukemia therapy, their efficacy and tolerability remain insufficient [13,40,41,42,43,44,45]. To address this, we developed the combined formulation of ASP and Dox in order to ensure a synergy effect through the use of “Aikido” micelles. The term “Aikido” in this context refers to the utilization of “smart” polymeric micelles for targeted delivery to tumors, leveraging their unique properties. The presented method has shown high efficiency and selectivity [29]. The expected advantages are the enhanced targetability, reduced hepatotoxicity, and cardiotoxicity, as well as optimized pharmacokinetics. Combinations of antitumor agents of ASP and Dox may exhibit a synergistic effect on cancer cells via different mechanisms, acting on distinct cellular targets, thereby reducing the required concentrations of each agent and minimizing the overall burden on the patient [40,46,47,48]. Each component in this formulation was optimized for maximum efficacy: ASP through conjugation with polymers [49] and Doxorubicin through the formation of polymeric “aikido” micelles [29].

The proposed “smart” dual-component formulation consists of an enzyme modified with cationic poly- or oligoamines that can form electrostatic complexes with the “smart” micelles, based on heparin, or chitosan grafted with oleic or lipoic acid residues. Heparin derivatives have the ability to suppress tumor growth and inhibit metastasis by inhibiting heparanase and P-/L-selectin activity at various stages. This formulation aims to harness the potential synergy between the enzyme and the cytotoxic drug to combat cancerous lymphoblastic cells. In this study, we investigate the catalytic activity and anticancer efficacy of EwA when used with other components as a part of smart micellar formulations, (L-asparagine-polycation) + (Doxorubicin micelles), compared to single components and untreated controls.

## 2. Materials and Methods

### 2.1. Reagents

The fluorescent substrate β-aspartic acid (7-amido-4-methylcoumarin) (Asp-AMC) and polymers and oligoamines for modification of asparaginases—chitosan lactate 5 kDa (Chit5), polyethylenimine 1.8 kDa (PEI1.8), heparin (low-molecular-weight fractions 12–14 kDa), spermine (sp), and 1-ethyl-3-(3-dimethylaminopropyl) carbodiimide (EDC)—were purchased from Sigma-Aldrich (St. Louis, MO, USA). Other reagents—N-hydroxysuccinimide (NHS), salts, acids, and buffer components—were produced by Reachim (Moscow, Russia).

### 2.2. Enzymes

*Erwinia carotovora* (EwA) asparaginases were obtained as described earlier [50]. The initial activity of asparaginases was checked by the standard method of circular dichroism spectroscopy on a J-815 CD spectrometer (Jasco, Tokyo, Japan) [51].

### 2.3. Synthesis and Characterization of Copolymers for Covalent Modification of Asparaginases

#### 2.3.1. Chit5-PEI

Chitosan was dissolved in 1 mM HCl solution (1 mg/mL), and then the pH was adjusted to 7 using 0.1 M phosphate buffer until the final chitosan concentration reached 0.5 mg/mL. Then, carbonyldiimidazole (100 mg/mL in DMSO) was added to the solution in an amount of 0.25 to 1 (mol/mol) by chitosan amino groups (approximately 30–35 glucosamine units per 5 kDa polymer chain). The mixture was incubated for 20 min at 40 °C. Then, a solution of PEI1.8 in PBS (5 mg/mL) was added drop by drop until the chitosan/polyethylenimine 1:1 mass ratio was reached. The mixture was incubated for 4 h at 40 °C. Purification was performed using dialysis (2 × 12 h, cut-off weight 6–8 kDa) against H_2_O. Characteristics of Chit5-PEI: each Chit chain contained an average of 2–3 PEI chains; the average molecular weight was 11 ± 2 kDa.

#### 2.3.2. PEI-g-PEG (Polyethylenimine-Polyethylene Glycol)

The branched PEI-g-PEG was acquired from Sigma-Aldrich (St. Louis, MO, USA). Polymer characteristics: PEI: PEG (1:15), branched PEI M_w_ 25 kDa, PEG M_n_ 5 kDa.

#### 2.3.3. Characterization of Copolymers

The degree of modification of chitosan by PEI1.8 was determined by spectrophotometric titration of chitosan by amino groups using 2,4,6-trinitrobenzenesulfonic acid forming a colored adduct with amino groups (absorption at 420 nm). Non-modified reagents were used as control samples.

**Figure 1:**
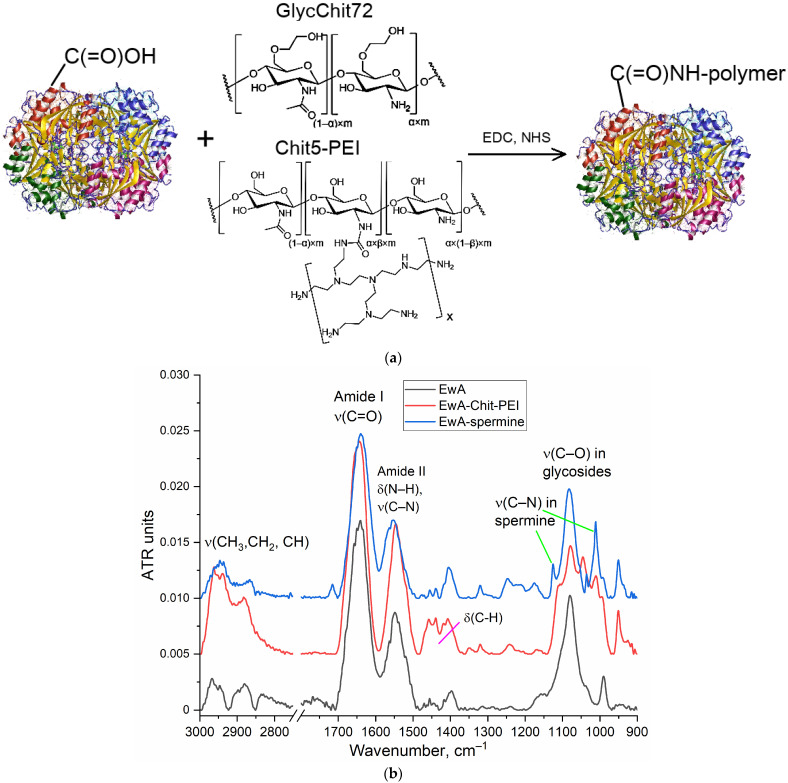
(**a**) The schemes of synthesis of asparaginase conjugates with Chit-PEI and GlycChit. (**b**) FTIR spectra of *Erwinia carotovora* L-asparaginase (EwA) in native form and modified by Chit-PEI, spermine. PBS (0.01 M, pH 7.4). T = 22 °C.

**Figure A1:**
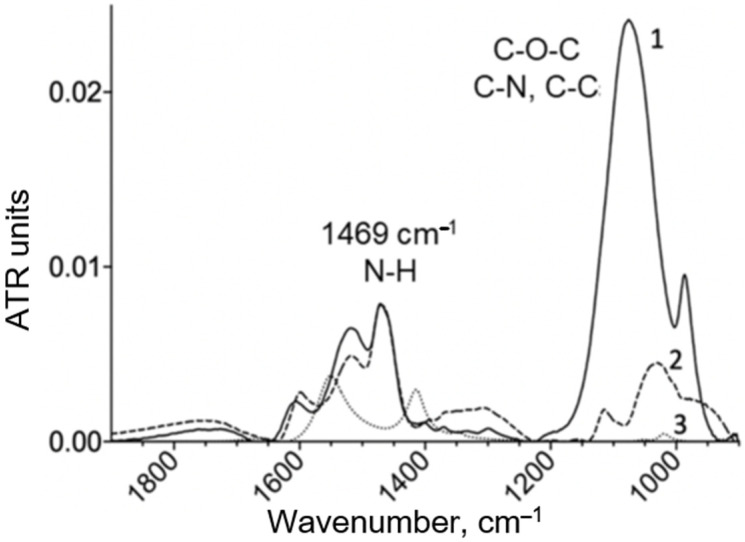
FTIR spectra of chitosan-PEI copolymer (1), PEI (2), chitosan 5 kDa 1 mg/mL (3) in 10 mM PBS pH 7.4.

The FTIR spectra of the copolymers were recorded using a MICRAN-3 IR microscope (Novosibirsk, Russia) and a Bruker Tensor 27 spectrometer (Bruker Optics, Ettlingen, Germany) equipped with a liquid-nitrogen-cooled MCT (mercury-cadmium telluride) detector. A characteristic high-intensity peak at 1000–1100 cm^−1^, which is highly intense (compared to chitosan), and peaks at 2950–2850 cm^−1^ were observed for PEI. Below is an example of the IR spectra (Figure 1 and Figure A1). The chemical structure of the copolymers was determined by the ratio of characteristic peaks for the initial components chitosan, PEI and spermine. Chemical crosslinking due to the formation of a urethane bond was confirmed by an increase in peak intensity at 1700–1600 cm^−1^ (ν(C=O)) and 1600–1500 cm^−1^ (δ(N–H)).

### 2.4. Synthesis and Characterization of Conjugates of EwA Asparaginases with Polymers and Oligoamines

Three polymers and one oligoamine were selected for the modification of EwA asparaginases (Figure S1): (1) sp(spermine); (2) PEI-g-PEG (polyethylenimine-polyethylene glycol); (3) Chit5-PEI (chitosan-polyethylenimine); (4) GlycChit72 (glycol-chitosan 72 kDa). Conjugate synthesis was performed using a carbodiimide approach.

The enzymes were dissolved in a phosphate buffer (0.02 M, pH 6.0) to a concentration of 1 mg/mL; then, a mixture of EDC and NHS (in CH_3_CN) was added in a mass ratio of 0.4 and 0.2 relative to asparaginase, followed by incubation for 30 min at 35 °C. Then, a solution of polymers (Chit5-PEI, spermine, PEI-g-PEG or GlycChit72) in the same buffer was added drop by drop to the activated enzyme solution until the following mass ratio was reached: enzyme–polymer = 1:0.3. The samples were incubated for another 2 h at a temperature of 35 °C. Purification was performed using dialysis (2 × 12 h, 4 °C, cut-off weight 12–14 kDa) against PBS. For additional purification and characterization of conjugates, HPLC chromatography was performed. The preparations were freeze-dried and then used in the work.

The conjugate mass was calculated based on the mass of the enzyme globule and the mass of the grafted polymer, the amount of which was determined spectrally (by FTIR).

The content of ASP and the number of copolymer chains in the resulting conjugates, and consequently, the ratio ASP-copolymer, were determined by CD and FTIR spectroscopy. The content of chitosan-PEI (or other copolymer studied) in the ASP conjugates was determined using the known modification degree of chitosan by PEI from the intensity of the characteristic absorption band of PEI at 1000–1100 cm^−1^ and 950 cm^−1^ in the IR spectrum (Figure A1), according to our previously developed method [36,37,38,39,40,41,42,43]. The concentration of the enzyme in the conjugates was controlled by CD spectroscopy.

### 2.5. Synthesis and Characterization of Amphiphilic Polymers and Micelles Containing Doxorubicin (Dox)

#### 2.5.1. Synthesis of Amphiphilic Conjugates Based on Heparin or Chitosan Grafted with Lipoic/Oleic Acid Residues

To obtain micelles, first, amphiphilic polymers based on polycations or polyanions and hydrophobic substituents were synthesized, as described in paper [29]. The synthesis schemes are shown in Figure S2. The idea of the synthesis was to activate the carboxyl group of lipoic (OA, conjugate—M1) or oleic acids (LA, conjugate—M2), followed by crosslinking with the chitosan amino group using a carbodiimide approach. In the case of heparin, the situation was reversed: the carboxyl group of heparin (Hep) and the amino group of oleylamine (M3) were crosslinked using the same approach. To obtain a conjugate of heparin and lipoic acid (M4) using a carbodiimide approach, spermine-modified (20% grafting degree) heparin was sequentially obtained, and lipoic acid residues were attached to Hep-sp via amino groups of spermine.

#### 2.5.2. Formation of M1–M4 Polymeric Micelles and Dox Loading—Non-Covalent Form

Conjugates Chit5-LA, Chit5-OA, Hep-sp-LA and Hep-OA were dissolved in PBS (0.01 M, pH 7.4) at a concentration of 10 mg/mL. Dox solution in PBS (2 mg/mL) was added to these samples at a mass ratio of 3:1 (polymer/Dox). For micelles formation and Dox, loading solutions were treated with probe-type ultra-sonic (snow, 10 min) followed by extrusion through a 200 nm membrane. Purification from unbound Dox was performed by dialysis for 6 h against PBS (cut-off 3 kDa). The final loading degrees are indicated in Section 3.2.

### 2.6. Characterization of Polymers, Micelles, Enzymes and Double Formulations

The FTIR spectra of the samples were recorded using a MICRAN-3 IR microscope and a Bruker Tensor 27 spectrometer equipped with a liquid-nitrogen-cooled MCT (mercury-cadmium telluride) detector.

Dynamic Light Scattering (DLS) was used to measure the particle sizes and zeta potentials using a Zetasizer Nano S “Malvern” (Worcestershire, UK).

Circular dichroism spectra were recorded on a Jasco J-815 CD spectrometer (JASCO, Tokyo, Japan) and used to assess the degree of Chit5 deacetylation, which was (92 ± 3)%.

Atomic force microscopy (NTEGRA II AFM microscope, NT-MDT Spectrum Instruments, Moscow, Russia) was used to visualize polymer micelles based on grafted chitosan and compare them in shape and size with unmodified chitosan.

### 2.7. Determination of the Dox Loading Degree in the Micelles

The amount of Dox contained in micellar preparations was determined by absorption at 490 nm and fluorescence intensity at 590 nm. UV spectra of solutions were recorded on the AmerSham Biosciences UltraSpec 2100 pro device (Cambridge, UK). Dox fluorescence was measured using a Varian Cary Eclipse spectrofluorometer (Agilent Technologies, Santa Clara, CA, USA) at 22 °C: λ_exci_ = 490 nm, λ_emi_ = 560 nm.

### 2.8. Determination of the Catalytic Activity of Asparaginase Formulations

#### 2.8.1. Fluorimetry

The enzyme (or conjugate) was added to a solution of 1 mM fluorescent substrate β-L-aspartate-(7-amido-4-methylcoumarin) (Asp-AMC) in PBS (0.01 M, pH 7.4) in the same buffer until a final concentration of 0.01–0.1 mg/mL was reached, intensively mixed, and then Kinetic curves were recorded: λ_exci_ = 360 nm, λ_emi_ = 465 nm. The activity was then determined along the initial linear section for 10 min (U/mg) in accordance with the standard EcA preparation.

#### 2.8.2. Circular Dichroism Spectroscopy (CD)

The measurements were performed on a Jasco J-815 CD device with a temperature-controlled cell. In a typical experiment, a solution of the enzyme (or conjugate) in PBS (0.01 M, pH 7.4) was added to the asparagine solution in the same buffer to a final concentration of asparagine 10 mM, enzyme 0.005–0.1 mg/mL (depending on specific activity). The reaction was carried out at a temperature of 37 °C, kinetic curves were recorded based on changes in ellipticity at a wavelength of 210 nm, and the Km and Kcat parameters were determined.

#### 2.8.3. Fourier Infrared Spectroscopy

IR spectra were recorded with a frequency of 1 min and a resolution of 2 cm^−1^ on a Bruker Tensor 27 device (Bruker, Ettlingen, Germany) equipped with an MCT detector with liquid nitrogen cooling and a thermostat (Huber, Raleigh, NC, USA). Additionally, 30-fold scanning and averaging were performed. In a typical experiment, a solution of the enzyme (or conjugate) in PBS (0.01 M, pH 7.4) was added to the asparagine solution in the same buffer until the final concentration of asparagine was 10 mM and the enzyme 0.005–0.1 mg/mL (depending on specific activity). The reaction was carried out at a temperature of 37 °C. The experiments were conducted under saturated conditions on the substrate, and therefore operated in a maximum rate regime.

### 2.9. Eukaryotic Cell Cultivation, Asparaginase and Dox Formulations Toxicity Studies

Raji lymphoblast-like cells were obtained from the collection of living systems of Lomonosov Moscow State University (Moscow, Russia). Eukaryotic cells were cultured in RPMI-1640 medium (Gibco, Thermo Fisher Scientific Inc., Waltham, MA, USA) with the addition of 5% fetal bovine serum (Capricorn Scientific, Ebsdorfergrund, Germany) and 1% Na-pyruvate (Paneco, Moscow, Russia) at 5% CO_2_/95% air in a humid atmosphere at a temperature of 37 °C. The toxicity of asparaginase preparations and its conjugates with polymers was determined using an MTT test [29].

The synergy coefficient K_syn_ = CV(X) × CV(Y)/(100 × CV(X + Y)), where CV is the cellular survival rate (in %) under the action of substances X, Y or a mixture of X + Y. If 0.95 < K_syn_ < 1.05, then additivity is observed; K_syn_ ≤ 0.95, then antagonism is observed; K_syn_ ≥ 1.05, then synergism is observed.

### 2.10. In Vivo Experiments

#### 2.10.1. Animals

Institutional Review Board Statement: The animal study protocol was approved by the Bioethics Committee at the Lomonosov Moscow State University no:110, 27 February 2020. The experiments were conducted in conjunction with MSU FFM on Wistar rats weighing (400 ± 20) g, which were kept under a 12 h light regime and a standard diet. Before the experiment, the rats were anesthetized and two catheters were implanted: one into the jugular vein to administer the drug, the second into the carotid artery to draw blood.

#### 2.10.2. Protocol of Experiments on the Pharmacokinetics of Doxorubicin and Accumulation of the Drug in Organs

The drugs were administered intravenously to animals 1 mL into the tail vein at a rate of 1 mg/mL per doxorubicin. Blood was taken into tubes with C-EDTA from the pineal vein after 5, 15, 45, 60 min, 2 h, 4 h, 8 h.

Then, 4–8 h after intravenous administration of the drugs, the animals were slightly anesthetized; after decapitation, the liver, kidneys and lungs were analyzed. Doxorubicin was extracted from blood and organ samples into methanol at the rate of 400 µL alcohol per 100 µL suspension/homogenate.

Quantitative analysis of Doxorubicin content in blood and organs using fluorescence spectroscopy (λ_exci_ = 490 nm, λ_emi_ = 600 nm). Fluorescence spectra were recorded using a SpectraMax M5 device (Downingtown, PA, USA) in the Costar black\clear bottom tablet (96 wells).

## 3. Results and Discussion

### 3.1. Strategy and Tactics of Work

In this work, the primary focus is the comparative analysis of native and polymer-modified ASP included in micellar formulations with Dox, in terms of its catalytic activity, and cytotoxic efficacy against cancer cells in vitro to reveal the conditions of synergistic effects (in combined forms versus individually) and to improve their pharmacokinetic properties.

A noteworthy aspect is the inclusion of ASP and Dox into micellar formulations based on heparin. Heparin has an antithrombotic effect, and the electrostatic interactions between the amine groups on the heparin and the drug molecules help to stabilize the formulation and thus to enhance the catalytic activity of the enzymes and the cytotoxic efficacy of the drugs.

In order to achieve these objectives, the following activities must be undertaken:

1. Synthesis and characterization of EwA asparaginase conjugates with polycationic polymers.
2. Study of Dox loading and its distribution in polymeric micelles based on chitosan or heparin, grafted with oleic/lipoic acid residues
3. Study of the influence of inclusion of different compositions in micellar formulations on catalytic activity of ASP forms (native, conjugated with cationic polymers, in combined with polymeric micelles) using FTIR spectroscopy and fluorimetry methods.
4. Cytotoxic efficacy of ASP conjugates and doxorubicin individually and in combination. Determination of the synergistic coefficients of cytotoxic action against Raji lymphoid cancer cells.
5. Pharmacokinetic characteristics of polymeric micelles formulations in vivo in rats.

This will achieve the development of biocompatible and highly effective formulations is an essential task to minimize immune responses and enhance the effectiveness of leukemia treatment.

### 3.2. Characteristics of Native and Polymer-Modified Asparaginases

Modification of ASP with cationic polymers was performed by the carbodiimide technique (the resulting conjugate schemes and synthesis schemes are shown in Figure 1a and Figure S1 in the Supplementary Materials). The covalent modification was based on the formation of an amide bond between the carboxyl group of the enzyme and the amino group of the oligomer or polymer. Confirmation of successful synthesis was performed using FTIR, as shown in Figure 1b. The following characteristic bands for the enzyme appear in the FTIR spectra: Amide I (ν(C=O), 1700–1600 cm^−1^); amide II (δ(N–H), ν (C–N), 1600–1500 cm^−1^); ν (C–H), 2980–2850 cm^−1^; and ν(C–O) in carbohydrate fragments, 1100–1000 cm^−1^. L-Asparaginase conjugates with oligo- and polycationic polymers are characterized in the FTIR spectra by a shift in the position of maxima for amide I (from 1645 to 1640 cm^−1^), amide II (from 1546 to 1553 cm^−1^), as well as the appearance of a ν(C–N) band due to fluctuations in the residues of spermine, chitosan or PEI that are manifested are in the range from 1250 to 1120 cm^−1^. The number of polymer chains in conjugates was determined based on the intensity of the band 1000–1030 cm^−1^ in the IR spectra of conjugates. The physico-chemical properties of native and modified EwA L-asparaginase, including data on asparaginase activity, are shown in Table 1. When modified with polymers or spermine, *K*_M_ practically did not change, and the activity increased by about 30% due to a shift in the pH of the optimum closer to the physiological values of 7.4. We also observed this effect for asparaginases from other sources [49,50,51].

### 3.3. Characterization of Amphiphilic Polymers and Micellar Formulations of Doxorubicin

**Figure 2:**
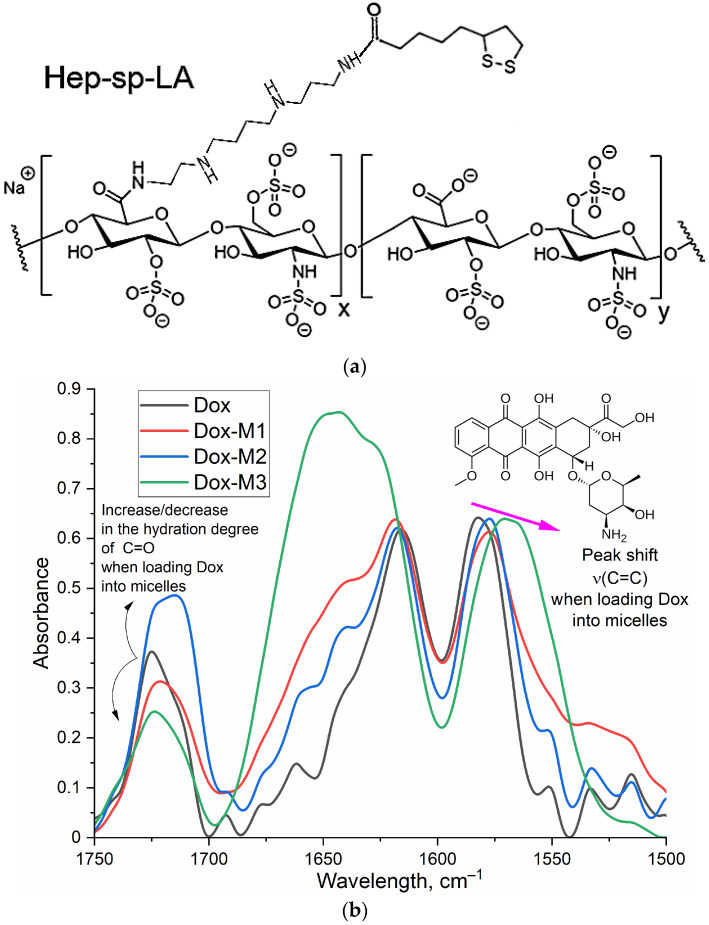

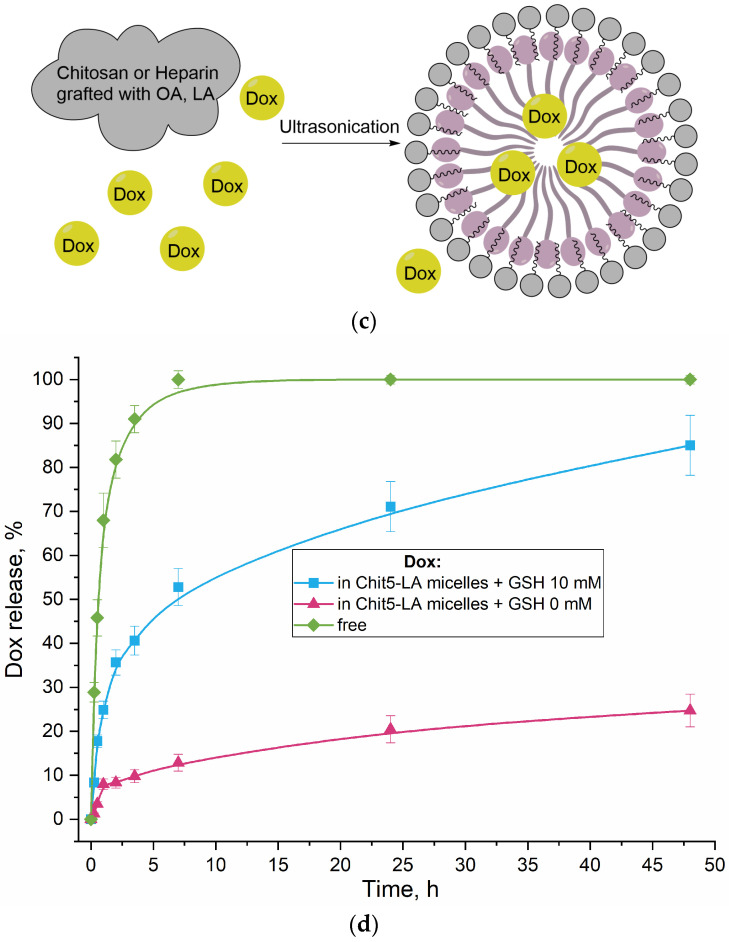
(**a**) The chemical structure of Hep-sp-LA (M3). (**b**) FTIR spectra of Doxorubicin (Dox) in free form and loaded into polymeric micelles M1–M3. PBS (0.01 M, pH 7.4). T = 37 °C. (**c**) The scheme of polymeric micelle preparation. (**d**) Dox kinetic release profiles: Dox free and Dox in Chit5-LA micelles in presence and absence of GSH as trigger (tumor microenvironment). The release was carried out in 10 mL of an external solution from 1 mL of a sample placed inside a dialysis cassette (6–8 kDa). PBS (0.01 M, pH 7.4). T = 37 °C.

To achieve targeted delivery of Doxorubicin to tumors, we applied “smart Aikido micelles” based on chitosan or heparin that were grafted with residues of oleic and lipoic acids. Variations in the hydrophobic tail in micelles were necessary to optimize the formulation’s structure and targeting properties: long oleic acid residues versus short lipoic acid tails; chemically inactive nature of oleic acids versus RedOx sensitivity of lipoic residues due to the S-S equilibrium shifting towards 2S-H (Figure 2a). To form a stable multipoint electrostatic complex with heparin (an anionic polymer), the ASP modified by polycations of different compositions were used. Chitosan functions as a cationic polymer able to form electrostatic complexes with carboxylic groups of ASP. To characterize the chemical composition and structure of polymeric micelles and Dox formulations, Fourier transform infrared (FTIR) spectroscopy was used, which provided crucial information for analyzing the molecular composition and chemical structure of biological polymers, which were the formulations studied.

Figure S3a presents the FTIR spectra of chitosan (Chit5), oleic acid (OA), and conjugates of Chit5 and OA. When chitosan is grafted with oleic acid, the formation of a C(=O)NH amide bond from the COOH results in a decrease in the intensity of a peak at 1710 cm^−1^, the appearance of two additional peaks at 1660 and 1560 cm^−1^ (Figure S3a), and a decrease in the intensity of the NH_2_ oscillation band (3600–3200 cm^−1^). The formation of micellar structures is confirmed by a change in the shape of the peak corresponding to C–O–C oscillations in chitosan glycoside residues due to the formation of hydrophobic and hydrophilic sites in micelles and a change in the conformation of chitosan macromolecules.

In the FTIR spectra (Figure S3b) of heparin (Hep) and its conjugates, intense bands corresponding to S=O stretching in SO_3_^−^ (1250 cm^−1^) and a C–O–C stretching band (1100–1000 cm^−1^) are observed. The grafting of Hep with LA or OA residues results in a the formation of an amide C(=O)NH bond, which is reflected in the IR spectrum as the appearance of absorption bands of Amide 1 and 2. Conjugate spectra contain absorption bands characteristic of both the initial components and the functional groups formed during crosslinking.

The DOX loading into polymeric micelles is driven by hydrophobic interaction (Figure 2b). Indeed, in the FTIR spectra of Dox there is a shift towards the lower frequency region of the oscillation band of C=C bond in the aromatic core of the doxorubicin molecule, with a change in intensity at 1720–1740 cm^−1^, which suggests a reduction in the hydration level of carbonyl groups in doxorubicin when it is incorporated into M2 micelles (where hydrophobic oleic acid residues form the core). Conversely, the hydration state of carbonyl groups is increased in doxorubicin in M1 and M3 formulations (where lipoic acid residues and partially charged polymeric groups form the core) (Table 2). The largest differences in FTIR spectra (Figure 2b) are observed for M3 micelles (heparin-lipoic acid). Therefore, in M1 and in M3 micelles, Dox has a more hydrophilic microenvironment than in M2, and most probably, Dox is located at the at the interface: the hydrophobic core—a hydrophilic surface.

Additionally, the amount of doxorubicin that can be loaded into the micelles varies by 18–22% in non-covalently bound micelles when adding 25 mass.% Dox. The degree of incorporation of Dox into heparin-based micelles is slightly lower than that of chitosan-based micelles, although both values are still within a similar range. This difference may be attributed to the greater hydrophobic nature of chitosan polymer chains compared to those of heparin.

The release of doxorubicin (Dox) from polymer micelles containing LA residues is stimulus-sensitive (Figure 2d). The trigger for the release is glutathione (GSH). Without a trigger, approximately 80% of the drug remains encapsulated in the micelles after two days, while in the presence of the trigger, Dox is released from the Chit5-LA micelle at the initial stage approximately 5 times faster, and the total amount of Dox accumulated after two days is 4 times higher. Therefore, polymer micelles provide selectivity for Dox in relation to the tumor microenvironment.

NMR spectroscopy was used for the characterization of Chit5 grafted with acids and to confirm the chemical conjugation. ^1^H NMR spectra of Chit5 and Chit5 grafted with LA and OA are presented in Figure S4. In the Chit5 ^1^H NMR spectra characteristic peaks (δ, ppm) are observed as follows [52,53,54,55,56]: 4.22 (H1), δ = 3.23 (H2), δ = 3.79, 3.96 (H3, H4, H5, H6, H6′), δ = 2.11 (NH–C(=O)–CH_3_). ^1^H NMR spectra of the Chit5-LA contained both signals of two components (chitosan and LA): 1.41 (S–H), increased signals at 2.0–2.3, and 1.25 were assigned to the methene hydrogen of the N-alkyl groups [57,58,59]. Signals of 3.64 ppm (C–H near the dithiolane fragment) and 2.3 ppm (β–H with relation to the carboxyl group) indicate the presence of LA in the conjugate [59]. Thus, NMR spectra confirm the structures of compounds described by the FTIR method.

Atomic force microscopy (AFM) was used to investigate the micelle-forming properties of the polymer conjugates under study. Figure S5 presents images of micelles formed by amphiphilic polymers. The mean size of Dox-M1 micelles was approximately 120–200 nm, while the size of DoxM2 micelles was around 130–180 nm. This discrepancy can be attributed to the fact that Dox-M2 contains more oleic acid residues compared to DoxM1, and the presence of lipoic acid in DoxM2 allows for the formation of disulfide bonds, which helps to stabilize the micelle structure.

Table 2 shows the resulting physico-chemical parameters of the synthesized amphiphilic conjugates: the grafting degree of chitosan per glycoside unit was 22–25%, and slightly less for heparin (due to the low content of carboxyl groups) at about 10–13%. At the same time, the average molecular weight of chitosan was 5 kDa, and its conjugates were 6–7 kDa. The average molecular weight of heparin was 12–14 kDa, and its conjugates were 13–15 kDa. Amphiphilic polymers were characterized by low values of critical micelle formation concentrations (CMC < 0.5 µg/mL) (Table 2); that is, micelles were formed even in a highly dilute solution, which determined potential stability in vivo.

**Table 2:** Physico-chemical properties of amphiphilic conjugates based on chitosan and heparin modified with oleic or lipoic acid residues. Parameters of doxorubicin-containing micellar formulations. PBS (0.01 M, pH 7.4). T = 37 °C.

**Amphiphilic Conjugates Based on Chitosan and Heparin**					
Designation *	Chemical formula **	The degree of polymer modification per glycoside unit, %	The average molecular weight of one structural unit, kDa	Zeta potential, mV	Critical Micelle Concentration (CMC) ***, µg/mL
M1	Chit5-LA	25 ± 3	6.4 ± 0.5	+12 ± 2	0.31 ± 0.04
M2	Chit5-OA	22 ± 4	6.8 ± 0.7	+15 ± 1	0.16 ± 0.05
M3	Hep-sp-LA	10 ± 2	14 ± 2	–10 ± 1.5	0.45 ± 0.05
M4	Hep-OA	13 ± 2	14 ± 2	–7 ± 1	0.28 ± 0.03
**Micellar compositions of Doxorubicin**					
Designation *			The mass content of Doxorubicin, %		
DoxM1			18 ± 2		
DoxM2			22 ± 3		
DoxM3			17 ± 1		
DoxM4			19 ± 2		

* M stands for micellar, MC stands for micellar covalent. ** Chit5—chitosan 5 kDa, Hep—heparin 12–14 kDa, LA—lipoic acid residue, OA—oleic acid residue, sp—linker spermine. *** Critical concentrations of micelle formation (CMC) were determined using a pyrene fluorescent probe [60].

**Figure 3:**
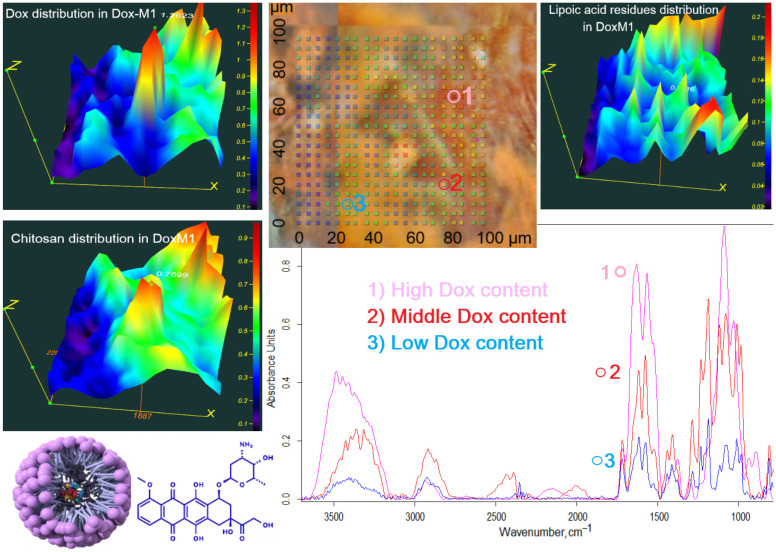
FTIR mapping of the DoxM1 sample (Dox in Chit5-LA polymeric micelles): integrated maps of the distribution of components over an area of 100 × 100 µm × µm: Dox (1680–1540 cm^−1^), Chit5 (1200–1000 cm^−1^) and LA (3000–2850 cm^−1^). In 3D graphics: red color corresponds to a high component content, blue to a low one. The scanning step is 5 µm. T = 22 °C.

An essential aspect of micellar formulation is the consistency (co-localization) of the component content and the incorporation of the drug molecule. A mapping of the DoxM1 sample was conducted using FTIR microscopy (Figure 3). The heat maps demonstrate the localization of the distribution of chitosan and doxorubicin within the micelle, indicating uniform loading of Dox: sites with a high chitosan content correspond to sites with a high Dox content. Conversely, the distribution of lipoic acid appears to be in the form of “forest”, indicating the formation of hydrophobic cores of micelles with Dox loaded inside. Therefore, by using FTIR microscopy, it is possible to examine the fine details of the structure of polymer particles. The formation of micellar structures has been demonstrated for the presented systems, which consist of hydrophilic regions and hydrophobic cores with loaded Dox.

### 3.4. Determination of the Catalytic Activity of Asparaginase Formulations

The idea of creating a combined formulation of L-Asparaginases + Doxorubicin suggests a possible increased effectiveness of each component compared to single drugs. For L-asparaginase, an important parameter is the catalytic activity, which is mainly increased by covalent conjugation with cationic polymers (Table 1), but it may be affected when they are included in the micellar formulation with the addition of polymeric micelles (due to a change in the microenvironment, stabilization or destabilization of the oligomeric structure of the enzyme, or adsorption at the interface of phases in micelles). Therefore, it is important to optimize the composition of the combined formulation in terms of its high enzymatic activity. So, the inclusion of ASP modified by polycationic polymers in the micellar system with negatively charged heparin-based micelles is driven by electrostatic interactions. Additionally, we investigated control samples, which include combinations of cationic chitosan micelles with ASP.

#### 3.4.1. Determination of the EwA Conjugates Activity by FTIR Spectroscopy

**Figure 4:**
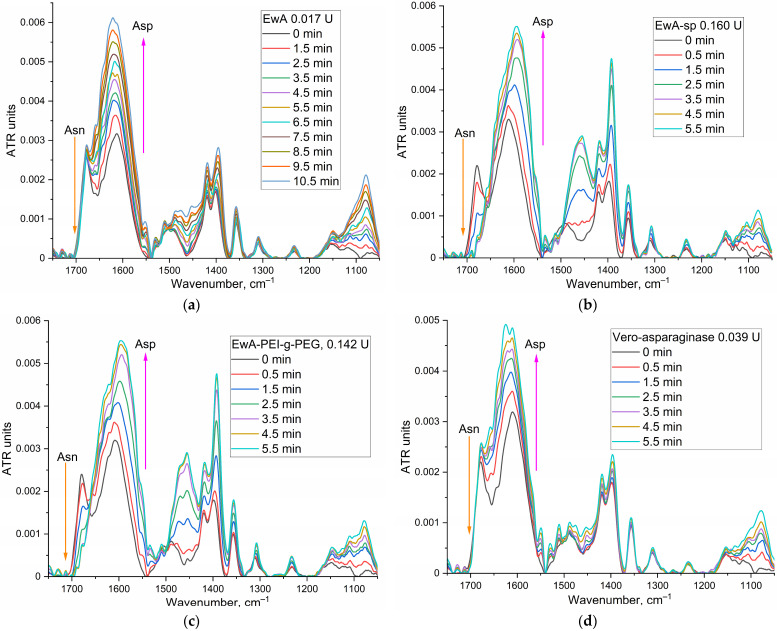

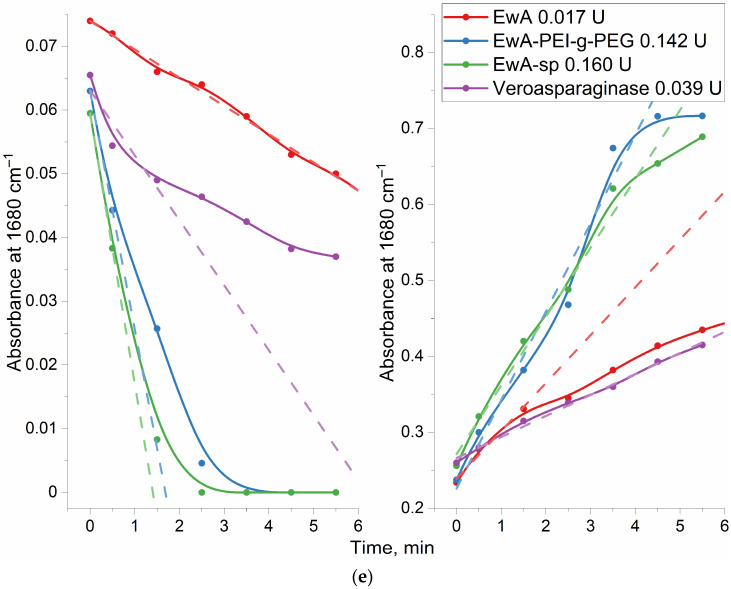
(**a**–**d**) FTIR spectra of asparagine (10 mM) during catalytic hydrolysis under the action of EwA and its conjugates, as well as the control medical enzyme Vero-asparaginase. Various activities (ME) are given to compare typical changes in the FTIR spectra. (**e**) Kinetic curves of hydrolysis of asparagine (10 mM) under the action of EwA and its conjugates, as well as the medical enzyme Vero-asparaginase. PBS (0.01 M, pH 7.4). T = 37 °C. Dotted lines indicate tangents to the initial section.

To determine asparaginase activity by FTIR spectroscopy, Asn concentration of at least 5–10 mM is required to quantify activity. It is necessary to select the experimental conditions so that the reaction proceeds by 80% in 3–10 min in order to register the initial section and, at the same time, so that there are no serious background distortions of the system. Figure 4 shows examples of FTIR spectra for the reaction of catalytic hydrolysis of L-asparagine under the action of EwA and its derivatives, as well as the medical commercial enzyme preparation Vero-asparaginase (as a control drug with known activity). Analytically significant are the peaks corresponding to symmetric valence vibrations ν_s_(C=O) in the molecules of the substrate (1680 cm^−1^) and the product (1610 cm^−1^). Peaks of 1450–1380 (ν_as_(COO^−^)) cm^−1^ and 1080 cm^−1^ (ν(C–O)) can also be considered; however, in the case of modified asparaginases, these parameters can give a serious error. Therefore, the main parameter is the substrate peak at 1680 cm^−1^. Figure 4e shows the kinetic curves corresponding to the spectra shown in Figure 4a–d. Based on the initial linear section of the curve, the maximum specific activity of enzymes was determined (Table 1).

#### 3.4.2. Determination of the EwA Activity in the Micellar Formulations by Fluorimetry

**Figure 5:**
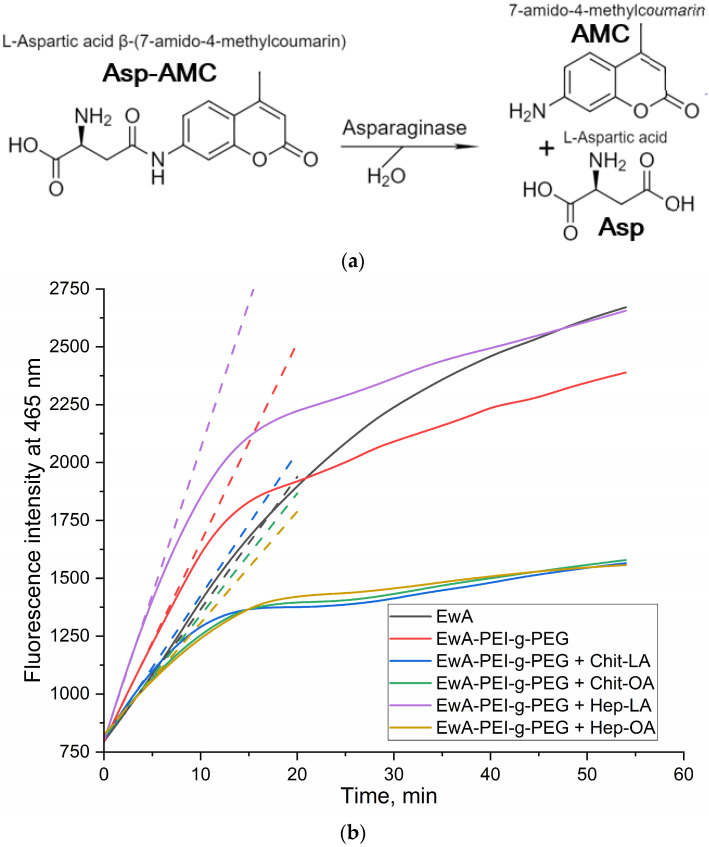
Fluorometric determination of the activity of EwA formulations: (**a**) enzyme-catalyzed reaction; (**b**) examples of kinetic curves of hydrolysis of Asp-AMC (2 mM) to Asp and AMC in the presence of 0.01 mg/mL EwA or EwA-PEI-g-PEG conjugate in combination with polymeric micelles M1-M4 (1:1 *w*/*w*) (Table 2). PBS (0.01 M, pH 7.4). T = 37 °C. Dotted lines indicate tangents to the initial section.

The fluorescent substrate Asp-AMC, aspartate amide and 7-amino-4-methylcoumarin are used for the fluorometric determination of asparaginase activity for micellar systems (Figure 5a). Based on the initial linear interval of the kinetic curves, the values of enzyme activity can be calculated (Figure 5b). For native EwA, the activity under saturating conditions is 350 ± 30 IU/mg, while the EwA-PEG-g-PEI conjugate is more active—490 IU/mg. Combined formulations (EwA-PEG-g-PEI + polymer micelles) are less active than the native enzyme ~310–340 IU/mg (chitosan based M1, M2, and heparin based M4), except for one leading sample: EwA-PEG-g-PEI + M3 micelles (Hep-LA), which demonstrate the maximal activity of 680 ± 70 IU/mg. This is achieved due to the formation of electrostatic complexes of heparin with cationic polyethylenimine. And chitosan analogous (Chit5-LA, M1) affects the activity of EwA conjugates negatively due to electrostatic repulsion. At the same time, oleic acid seems to have a negative effect on the catalytic properties of the enzyme. Lipoic acid is shorter than oleic acid, and therefore creates a less hydrophobic environment, and in addition, LA is characterized by RedOx sensitivity, which, conversely, can improve the biocatalytic properties of the enzyme. The structure of micelles derived from chitosan or heparin modified with lipoic acid moieties is optimally suited for combination with EwA due to its enhanced hydrophilicity and favorable polymer conformation.

Table 3 shows the resulting activities of EwA formulations: native enzyme, conjugates with polymers and combined with polymeric micelles. The best activity was demonstrated by EwA samples in combination with M3 (Hep-LA) micelles, since heparin is a polyanion and can electrostatically affect the enzyme. OA residues (for both polymers, heparin and chitosan) appear to have a negative effect on the enzyme. The leading formulations are EwA-PEI-g-PEG and EwA-GlycChit in combination with M3, probably due to the optimal density of the positive charge.

### 3.5. Cytotoxic Effect of Dox and ASP Components Individually and in Combination against Raji Cancer Cells: MTT Analysis and CLSM Visualization

**Figure 6:**
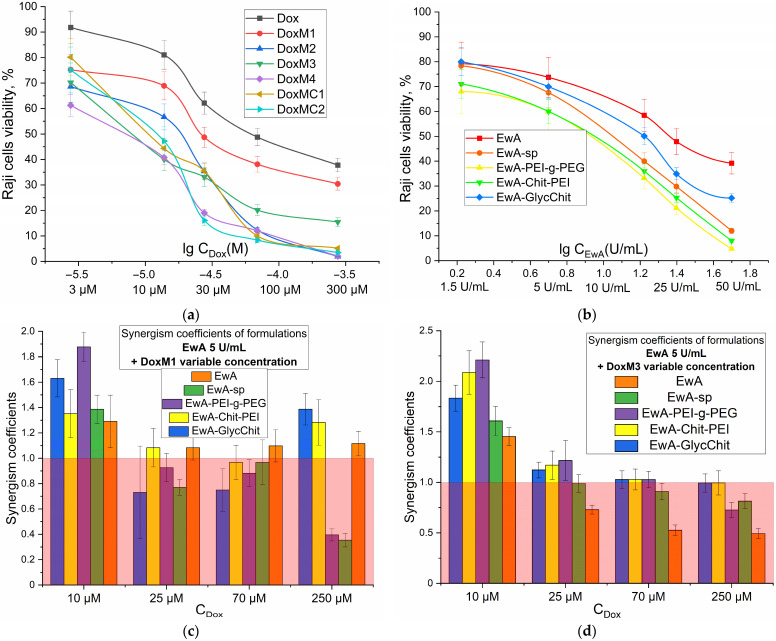
MTT analysis of the viability of Raji cells under the action of: (**a**) Doxorubicin and its micellar formulations; (**b**) EwA and its conjugates with polymers; (**c**) the combined formulation of DoxM1 + EwA and its conjugates with polymers; (**d**) the combined formulation of DoxM4 + EwA and its conjugates with polymers. RPMI-1640 medium with the addition of 5% fetal bovine serum and 1% sodium pyruvate at 5% CO_2_/95% air in a humidified atmosphere at a temperature of 37 °C. The red area corresponds to an inefficient combination of components, and the unpainted area corresponds to an effective one. The synergy coefficient K_syn_ = CV(X) × CV(Y)/(100 × CV(X + Y)), where CV is the cellular survival rate (in %) under the action of substances X, Y or a mixture of X + Y. If 0.95 < K_syn_ < 1.05, then additivity is observed; K_syn_ ≤ 0.95, then antagonism is observed; K_syn_ ≥ 1.05, then synergism is observed.

A key factor in determining the therapeutic efficacy of an antitumor formulation is its cytotoxic action against cancer cells. Figure 6a,b show the dose dependence of survival for Raji cancer cells (Burkitt lymphoma) on the concentrations of added Dox and EwA, respectively. When Dox was administered in micellar form, there was a significant increase in its cytotoxic activity, with a 20–40% increase in survival compared to free Dox. This corresponds to an improvement in IC_50_ (concentration of half-inhibition of cell growth) of up to 10-fold, from approximately 70 μM for free Dox to approximately 7 μM for Dox in micellar form. The following is a ranked list of the cytotoxic efficacy of Dox preparations: Dox < DoxM1 < DoxM2 < DoxM3 < DoxMC1 <≈ DoxMC2 ≈ DoxM4. Therefore, heparin formulations are the most active and promising.

Similarly, for the ASP cytotoxic activity in the polymer-conjugated form is pronounced increased (by 10–50% in terms of cell viability), compared to the free form, and there is up to a 2.5-fold reduction in IC_50_ parameter from approximately 25 U/mg for EwA to 10 U/mg for EwA-PEI-g-PEG and EwA-chitosan-PEI. The following is a ranked list of the cytotoxic efficacy of EwA formulations: EwA < EwA-GlycChit < EwA-sp < EwA-Chit-PEG ≈ EwA-PEI-g-PEG.

For a combined formulation, the key parameter is the enhancement of the action of the components in the mixture compared to single formulations. For the formation of Dox and ASP, this can be achieved through the formation of electrostatic heparin-polyamine complexes, stabilization of the enzyme and enhanced permeability to cancer cells. The synergism coefficients (K_syn_) indicate how many times the survival rate of cells in the case of a combined formulation is less than the theoretical total effect of the individual components. Therefore, if 0.95 < K_syn_ < 1.05, additivity is observed; if K_syn_ ≤ 0.95, antagonism occurs; and if K_syn_ ≥ 1.05, synergism occurs.

Figure 6c,d illustrate the values of synergism coefficients for various combinations of L-asparaginase conjugates and micellar formulations of DoxM1 and DoxM4 at different Dox concentrations. In Figure 6c,d, the red region corresponds to an ineffective combination of components and the unshaded region corresponds to an efficient one. For DoxM1 (Dox non-covalent in chitosan-lipoic acid micelles), in combination with EwA-PEI-g-PEG, EwA-Chit-PEI or native enzyme, the formulations have shown to be effective across the entire concentration range (10–250 µM). In the case of DoxM3, a synergy is observed when combined with all EwA conjugates at Dox concentrations of 25 µM, since at higher concentrations, the components are quite active on their own. Therefore, among the formulations obtained, leading combinations EwA-PEI-g-PEG and EwA-Chit-PEI demonstrate synergistic effects up to two times greater (in terms of cell viability), which is a two-fold increase in activity when used in combination rather than individually. This offers promising prospects for the use of doxorubicin-asparaginase therapy in the treatment of leukemia.

To better understand the significance of our results, we compared them to other similar systems described in the literature. There are numerous combinations of Dox that have been described in the literature and are characterized by a synergistic effect. One such combination is that of camptothecin and doxorubicin [61], which has been shown in low-dose studies to exhibit synergism (with the potential for a 5-fold reduction in dosage) in several breast cancer cell line models in vitro. Other Dox combinations are also used to reduce dosages, increase efficacy, and reduce the toxicity of formulations: (i) gemcitabine and doxorubicin [62]; (ii) BH3-interacting domain death agonist (BID) protein + Dox [63]; (iii) Dox + Roscovitine [64]; and (iv) Dox + Resveratrol [65]. However, our findings are superior to those reported in the literature, as we have improved each component (Dox and ASP) and demonstrated the synergistic effects in the combination in micellar formulations. Consequently, the maximum cytotoxic effect of the Dox + ASP combination can be estimated to be 2.5 (enhancing EwA) × 10(enhancing Dox) × 2(synergy) = 50-fold higher than that of Dox or ASP alone.

We have thoroughly investigated the interaction of cells with various substances. One of the most informative methods for visualizing the effects of cytotoxic agents on cells is confocal laser scanning microscopy (CLSM), as shown in Figure S6. The figure presents fluorescent images of K562 cancer cells that were incubated with different doses of doxorubicin (Dox) preparations: micellar (non-covalently loaded Dox in Chit5-OA) and free Dox.

The best permeability was achieved for DoxM1, a micellar formulation of doxorubicin, while free doxorubicin showed relatively weak penetration into cells during a 2 h incubation period. The use of polymer micelles allows for selective delivery of doxorubicin specifically to cancer cells, as compared to normal cells (see Table 4 for details).

### 3.6. Pharmacokinetic Parameters of the Doxorubicin Component—The Effect of Polymer Micelles on the Properties of Antitumor Formation and Bio-Distribution

It is important to understand the influence of the micellar formulation on the pharmacokinetic parameters of the drug (prolonged action, increase in effectiveness), and to reduce the side effect (high dosages, hepatotoxicity and cardiotoxicity), which is realized due to the “Aikido” micelles M1–M4. In other words, Aikido micelles prolong the activity of Dox and ASP, prevent thrombosis, reduce the viscosity of the microenvironment around cancer cells, and thus facilitate the delivery of combination therapies.

**Figure 7:**
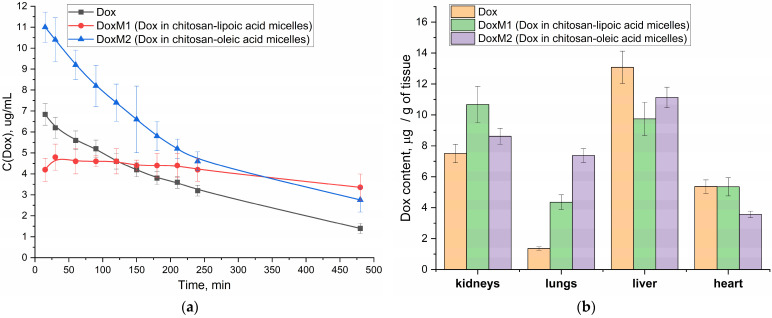
(**a**) Pharmacokinetic curves of Doxorubicin in free form and micellar (M1 and M2) in Wistar rats. (**b**) Bio-distribution of Doxorubicin in free form and micellar (M1 and M2) in the organs of Wistar rats.

Figure 7a shows the pharmacokinetic curves of Dox in free form and micellar (M1 and M2) in vivo experiments in Wistar rats. The initial (apparent) concentration of Dox in the micellar formulation of chitosan M1 is higher than that of free Dox and for M2, which is probably due to the initial sharp release of the drug from the surface layer of micelles (and further Dox is released gradually from the hydrophobic core). In both cases, in the case of M1 and M2 micelles, we observe that both parameters area under curve (AUC) and half-elimination time are increased compared to Dox free (Table 5).

For DoxM2 (chitosan-oleic acid), an increase in the area under the pharmacokinetic curve is more pronounced (Table 5), which is due to an increase in the effective concentration of Dox in the blood. For DoxM1 (chitosan-lipoic acid), a different effect is achieved: the concentration of Dox is maintained almost constant at 4–5 µg/mL for more than 8 h (Table 5), which is important from the point of view of the effectiveness and prolongation of the cytotoxic agent, provided that its concentration is retained in the therapeutic window. In the case of M1 formulations, it is likely that a different mechanism is employed, which can be described by a two-compartment model involving absorption due to the specific distribution of polymer micelles within the body. A steady-state concentration of Dox is observed rather than a rapid release.

In the practical point of view, DoxM1 particles in the blood are stable due to covalent crosslinking of the lipoic acid residues. At the same time, when the micelles enter the cancer cells, then the micelles will release the drug (Figure 2d)—this is the key difference. At the same time, the control micelles M2 with oleic acid are non–covalent particles formed mainly due to hydrophobic interactions—they provide higher AUC values (Table 5), but at the same time, clearance is higher than in the case of M1 micelles circulating in the bloodstream for a long time.

In addition, for micellar formulations, the bio-distribution of Dox to the liver and heart is reduced, which will reduce potential cardio and hepatotoxicity (Figure 7b).

It is important to note that micellar formations are characterized by a prolonged effect, and they will have a prolonged effect on both of the drugs in the bloodstream due to the formation of stable multipoint electrostatic complexes with the enzyme (since the binding constants of interpolyelectrolyte complexes are estimated at 10^6^–10^9^ M^−1^ [66]). In addition, polymers shield the immunogenic epitopes of the enzyme and make it “invisible” (stealth technology) to the immune system and thus safe. Thus, improved pharmacokinetic parameters of Doxorubicin in vivo have been shown, which plays a key role in creating safe and effective antitumor compositions.

### 3.7. Discussion of the Advantages of the Presented Combined Formula ASP + Dox

“Smart” drug delivery systems are currently being actively developed. It can be classified by carrier type [67]: polymeric, micelles, liposomes, dendrimers, etc. The delivery systems can be classified according to their mechanism of action:

- Target-specific nanocarrier delivery systems:

  Active targeting;

  Passive targeting;

- Intracellular delivery and sub-cellular distribution;
- Stimuli-responsive delivery:

  pH-responsive delivery;

  Temperature-responsive delivery;

  RedOx-responsive delivery.

All these drug delivery systems have their benefits. The ASP + Dox combined formulation suggested here is characterized by a significant advantage—the synergy of the components—which has not been previously presented. So, the advantages of our systems include the following:

Combined formulation of two antitumor drug, Dox and ASP. Therefore, a synergistic effect is achieved through the interaction of two distinct mechanisms (asparagine depletion and intercalation of doxorubicin into DNA), while both drugs within the combined delivery system selectively and simultaneously enter cancer cells. Thus, the combo formation turned out to be highly effective due to the synergy of the components and due to the strengthening of each component separately.

We used “smart Aikido micelles” to provide selective drug delivery, which is triggered by the breaking of the S-S bond mainly within cancer cells. These smart micelles exhibit increased cancer cells permeability, pronounced enhanced cytotoxic effects, and high selectivity index towards cancer cells in relation to healthy cells.

We proposed improved ASP micellar formulations (stabilized by electrostatic interactions). The expected effects of this approach include reduced immunogenicity due to shielding the protein surface with a polymer coating; increased enzyme activity and stability; and selectivity for tumor cells due to slight acidic microenvironment as well as the well-known effect of higher permeability and retaining of larger particles.

## 4. Conclusions

The paper presents the concept of developing a combined anti-leukemia treatment that combines two components: asparaginase (ASP) and doxorubicin (Dox). Four covalent conjugates of *Erwinia carotovora* asparaginase (EwA) with poly- and oligoamines were synthesized to improve biocatalytic properties and reduce immunogenicity. The activity of ASP conjugates compared with native enzymes was increased up to 1.5–2 times due to a shift in the pH optimum closer to physiological values and stabilization of the more active form of the enzyme: from 350 to 410–490 U/mg. Four non-covalent micellar formulations of doxorubicin, based on chitosan and heparin, modified with oleic and lipoid acid residues, were synthesized and analyzed, as well as two covalently bound prodrugs of doxorubicin and polymer with a labile S-S bond. A combined formulation of ASP and Doxorubicin was tested for catalytic activity using FTIR spectroscopy and fluorimetry: EwA conjugated with polymers + M3 micelles (Hep-LA) demonstrated the best catalytic activity, with values of 550–680 U/mg, respectively. Micellar Doxorubicin (non-covalent) is almost an order of magnitude more effective in terms of IC_50_ on Raji cells compared to free Dox. For EwA, conjugation reduces the IC_50_ on Raji cells by 2.5 times from 25 to 10 U/mg. In addition, combined formulations of ASP + Dox demonstrate synergism of up to two times (in terms of cell viability) enhanced paired activity. Improved pharmacokinetic parameters for Doxorubicin in the micellar formulation compared to unmodified Doxorubicin have been demonstrated, with an increase in effective concentration and prolonged activity lasting for more than 8 h.

Heparin and lipoic acid are essential components of the combined anti-leukemia treatment. Heparin helps prevent local thrombosis in tumor tissues, increasing the availability of cancer cells to the drug formulation. It also forms electrostatic complexes with cationic-modified ASP, enhancing the efficacy of the drug and improving its stability. Lipoic acid plays a crucial role in forming hydrophobic micelle cores for loading cytotoxic agents such as Dox. It also exhibits RedOx sensitivity, which is beneficial in targeting cancer cells and enhancing the effectiveness of Dox when combined with ASP in the Hep-LA formulation. These findings provide a basis for the development of novel, effective antitumor formulations.

## Figures and Tables

**Table 1:** Physico-chemical properties of native and modified asparaginases EwA. L-Asparaginase activity and *K*_M_ values were determined by fluorescence spectroscopy at 37 °C in PBS (0.01 M, pH 7.4). [S]_0_ = 1 mM.

Enzyme or Conjugate	Polymer/Protein Ratio, mol/mol	Zeta Potential, mV	Molecular Weight, kDa			*K*_M_ *, mM	k_cat_, s^−1^	ASP Activity *, U/mg
			Protein	Polymer/ Oligoamine	In Total			
EwA	-	0 ± 1	34 × 4 = 136	-	136	0.015 ± 0.004	2.6 ± 0.3	350 ± 30
EwA-sp	130 ± 20	2 ± 0.5		0.202	158–166		3.6 ± 0.4	490 ± 50
EwA-PEI-g-PEG	1.1 ± 0.1	8 ± 1		30 ± 5	160–172		3.6 ± 0.4	490 ± 60
EwA-Chit5-PEI	3.2 ± 0.5	15 ± 2		12 ± 2	163–188		3.2 ± 0.5	440 ± 60
EwA-GlycChit72	0.6 ± 0.03	7 ± 1		72 ± 10	330–360		3.0 ± 0.3	410 ± 30

* Confidence intervals are indicated at *p* = 0.05, calculated according to Student’s criterion.

**Table 3:** Values of the maximum specific activity (U/mg) of EwA formulations (native enzyme, conjugates with polymers and combined with polymeric micelles). C_Asn_ = 10 mM. PBS (0.01 M, pH 7.4). T = 37 °C. The color scheme corresponds to a change in activity relative to the “no additives” line: the darker the green, the higher the activity; the brighter the shades of red, the lower the activity; yellow corresponds to activity comparable to native EwA.

V_max_, U/mg	EwA	EwA-sp	EwA-PEI-g-PEG	EwA-Chit-PEI	EwA-GlycChit
**no additives**	350 ± 30	490 ± 50	490 ± 60	440 ± 60	410 ± 30
**+Chit-LA (M1)**	300 ± 10	260 ± 70	340 ± 50	370 ± 50	300 ± 30
**+Chit-OA (M2)**	290 ± 20	250 ± 20	315 ± 10	260 ± 18	350 ± 20
**+Hep-LA (M3)**	375 ± 10	555 ± 70	680 ± 70	380 ± 65	550 ± 30
**+Hep-OA (M4)**	290 ± 10	360 ± 70	310 ± 70	280 ± 65	380 ± 30

**Table 4:** The resulting schematic characteristics of Dox-containing formulations based on polymeric micelles in terms of tumor targeting. “++” means a bright effect, “+” means a good effect, “±” means a weak effect, “–” there is no effect.

Micellar Composition Containing Dox	Permeability to Eukaryotic Cells				Toxicity to Eukaryotic Cells		Tumor Targeting	
	Cancer K562	Cancer Raji	Cancer A875	Normal HEK293T	Cancer K562	Normal HEK293T	pH 5.5–6.5	Glutathione
Dox free	+	+	±	+	+	+	–	–
DoxM1 (Dox in Chit5-LA)	++	±	+	±	++	±	+	+
DoxM2 (Dox in Chit5-OA)	+	+	+	±	++	±	+	–

**Table 5:** Pharmacokinetic parameters of Dox in free and micellar form in Wistar rats.

Dox Formulation	Half-Elimination Time, min	Area under Curve 0–480 min (~Effective Concentration)
Dox free	220	800
DoxM1	>500	960
DoxM2	250	1270

## Data Availability

The data presented in this study are available in the main text and Supplementary Materials.

## Supplementary Materials

The following supporting information can be downloaded at: https://www.mdpi.com/article/10.3390/polym16152132/s1, Figure S1: The scheme of asparaginase conjugation with oligo- and polymers. The synthesis conditions are given in the methods section; Figure S2: The synthesis schemes of: (a) amphiphilic conjugates Chit5-LA (M1), Chit5-OA (M2) and Hep-OA (M4); (b) amphiphilic conjugate Hep-sp-LA (M3); Figure S3: FTIR spectra of: (a) Chit5 (chitosan 5 kDa), OA (oleic acid), and Chit5-OA conjugate; (b) Hep (heparin) and its conjugate Hep-OA. PBS (0.01 M, pH 7.4). T = 22 °C; Figure S4: ^1^H NMR of (a) Chit5, (b) Chit5-LA, (c) Chit5-OA. D_2_O. T = 25 °C; Figure S5: AFM images of polymeric particles (micelles) self-assembled from (a) DoxM1 and (b) DoxM2. (b), (d) The corresponding height profiles of (a,c). The surface is freshly ground mica; Figure S6: Fluorescence images of K562 cells after 2 h incubation with Dox-containing formulations. C_Dox_ = 2 μM. Dox channel: λ_exci_ = 500–560 nm, λ_exci_ = 590–700 nm. The scale segment is 100 µm. DoxM1—Dox in Chit5-LA.
