# Supplementary material for: Targeted Polymeric Micelles System, Designed to Carry a Combined Cargo of L-Asparaginase and Doxorubicin, Shows Vast Improvement in Cytotoxic Efficacy
Source: Polymers (Basel). 2024 Jul 26;16(15):2132. doi: 10.3390/polym16152132 (PMC11314107; doi:10.3390/polym16152132)

Targeted polymeric micelles system, designed to carry a combined cargo of L-asparaginase and Doxorubicin, shows vast improvement in cytotoxic efficacy

Igor D. Zlotnikov [1] and Elena V. Kudryashova [1,*]

[1] Faculty of Chemistry, Lomonosov Moscow State University, Leninskie Gory, 1/3, 119991 Moscow, Russia; zlotnikovid@my.msu.ru (I.D.Z.);

* Correspondence: helenakoudriachova@yandex.ru

Figure S1. The scheme of asparaginase conjugation with oligo- and polymers. The synthesis conditions are given in the methods section.

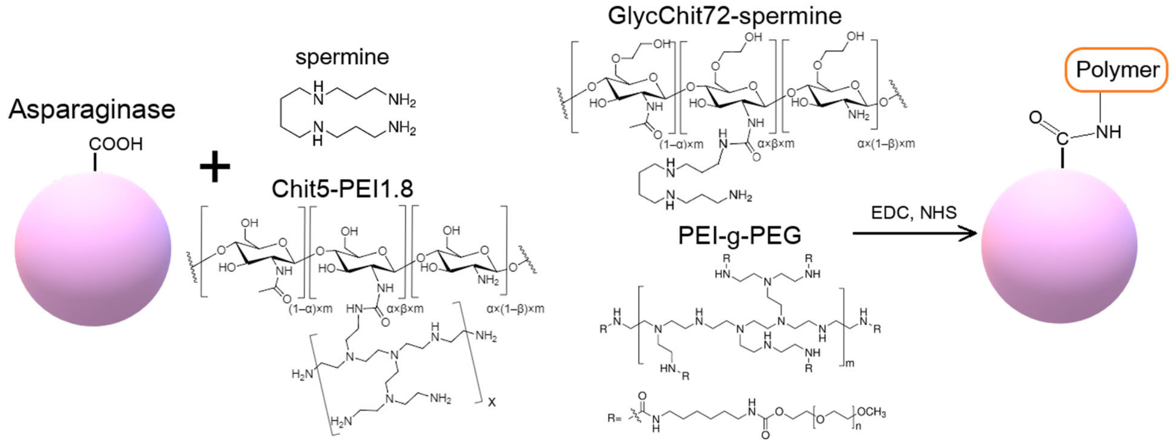

Fig. S2. The synthesis schemes of: (a) amphiphilic conjugates Chit5-LA (M1), Chit5-OA (M2) and Hep-OA (M4); (b) amphiphilic conjugate Hep-sp-LA (M3); [10.3390/gels10030157].

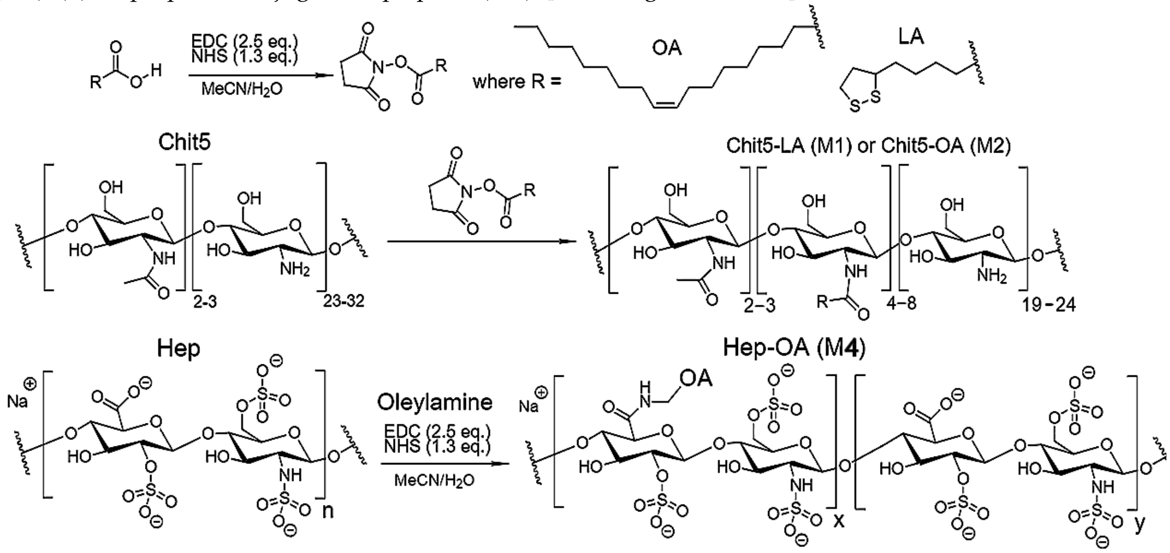

(a)

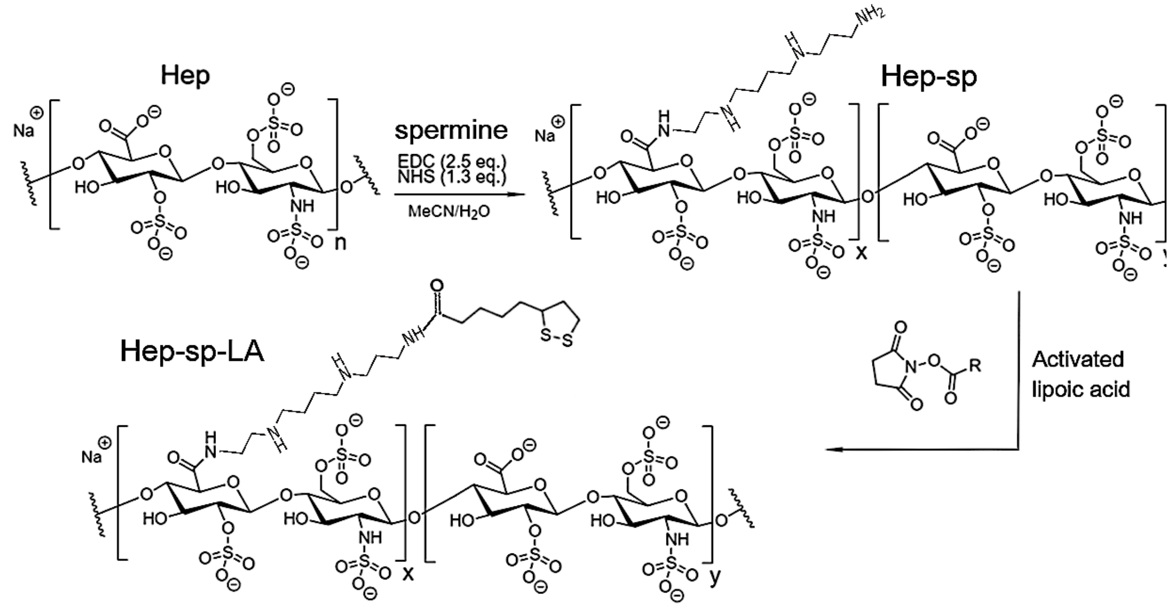

(b)

Fig. S3. FTIR spectra of: (a) Chit5 (chitosan 5 kDa), OA (oleic acid), and Chit5-OA conjugate; (b) Hep (heparin) and its conjugate Hep-OA with oleylamine residues. PBS (0.01 M, pH 7.4). T = 22 °C.

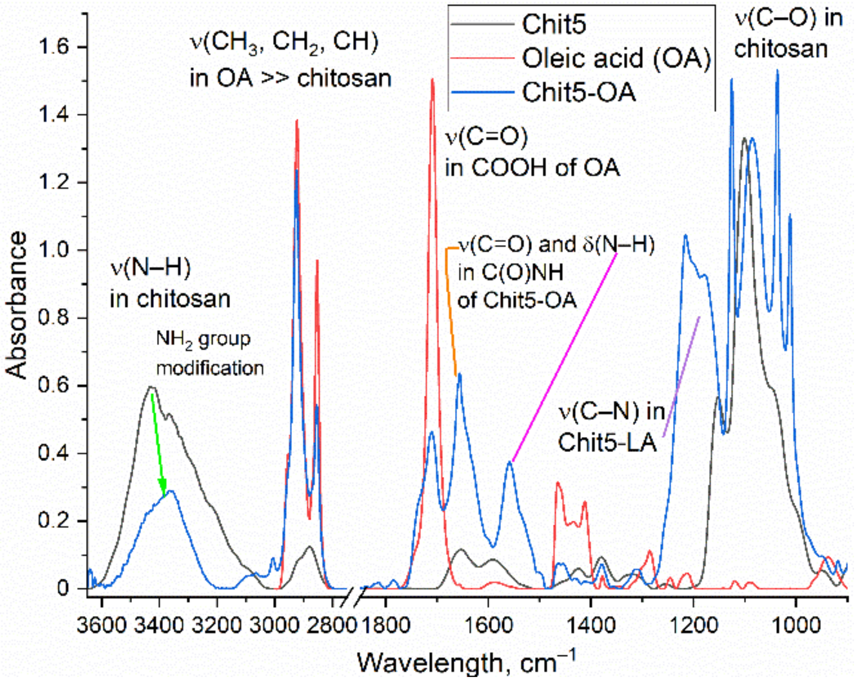

(a)

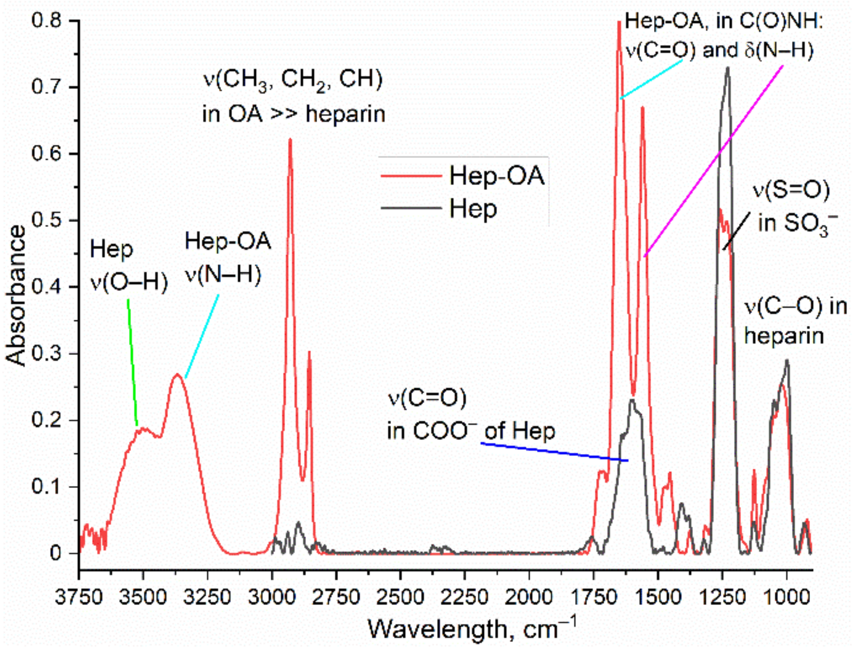

(b)

Fig. S4. ^1H NMR of (a) Chit5, (b) Chit5-LA, (c) Chit5-OA. D_2O. T = 25 °C.

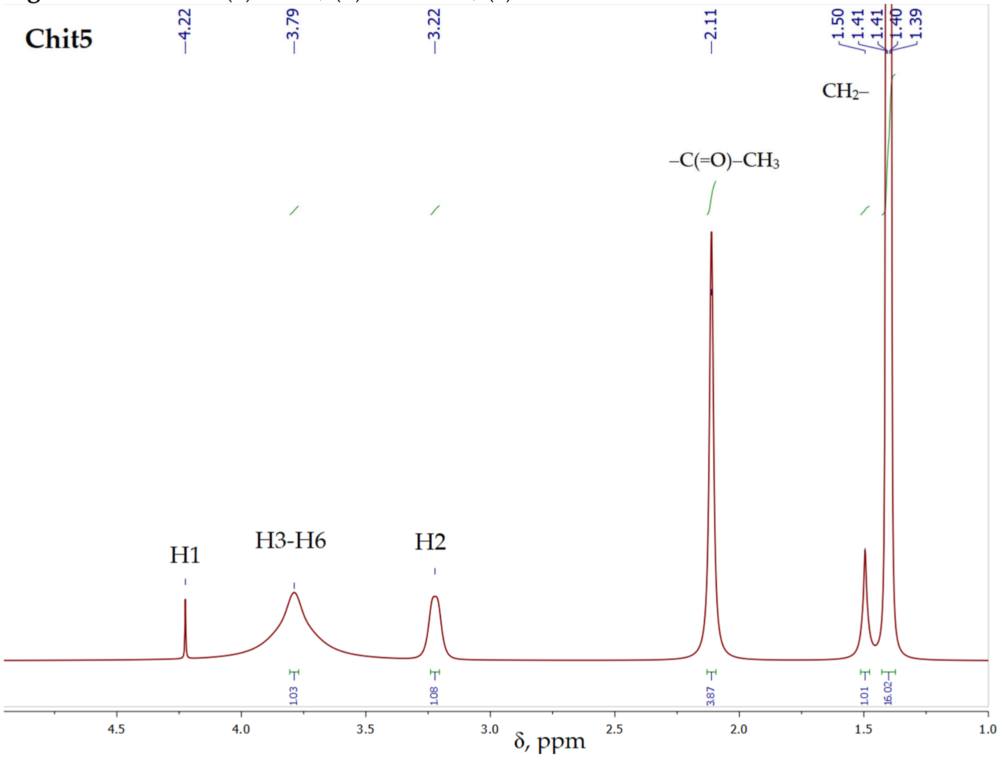

(a)

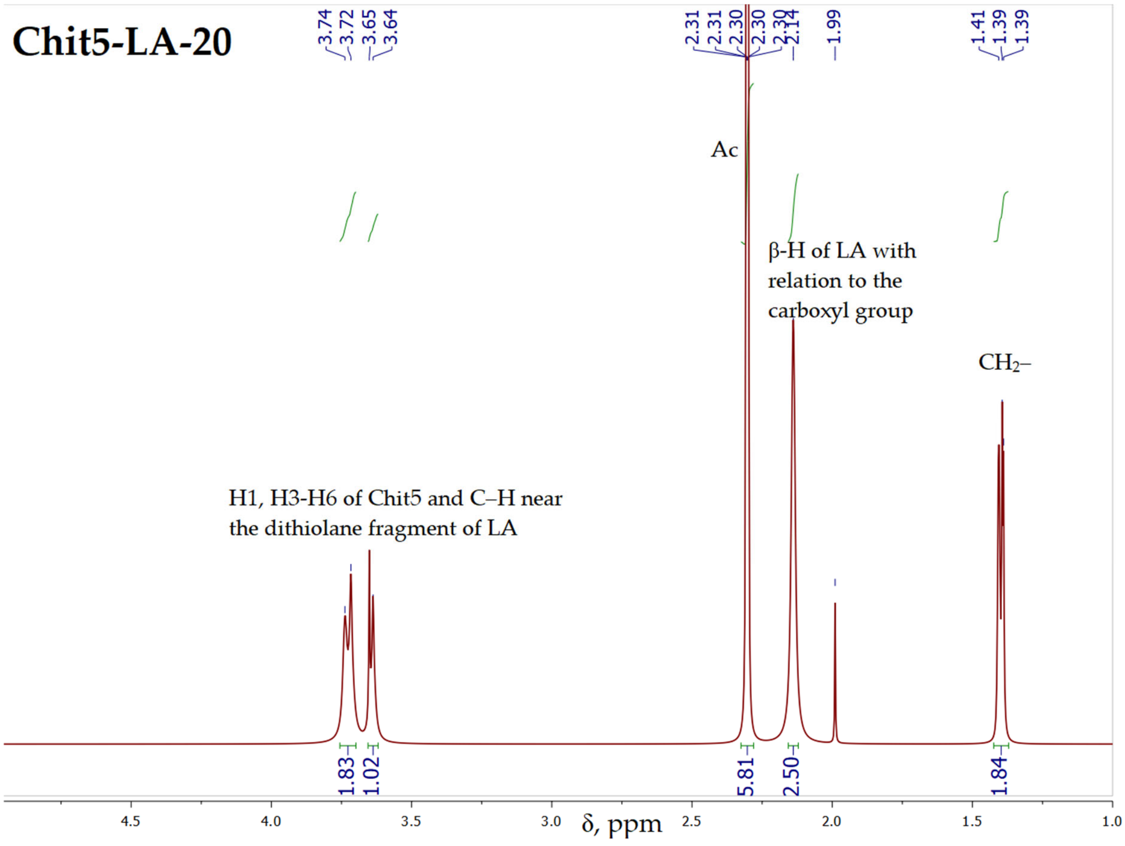

(b)

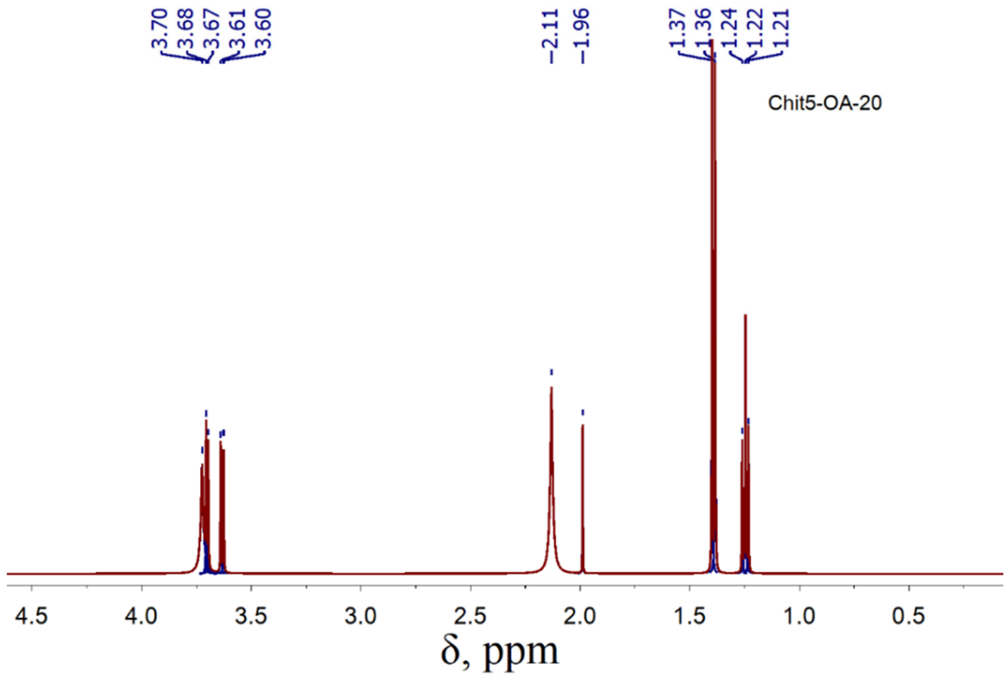
3.70
3.68
3.67
3.61
3.60
2.11
1.96
1.37
1.36
1.24
1.22
1.21
Chit5-OA-20
4.5
4.0
3.5
3.0
2.5
2.0
1.5
1.0
0.5
δ, ppm

(c)

Figure S5. Atomic force images of micelles formed from (**a**) DoxM1 and (**c**) DoxM2 conjugates. (**b**), (**d**) The corresponding height profiles of (**a**,**c**). The surface is freshly ground mica.

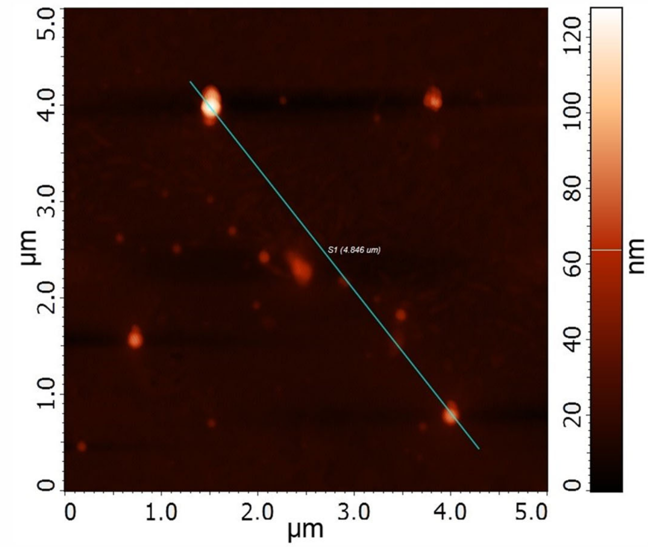

(**a**)

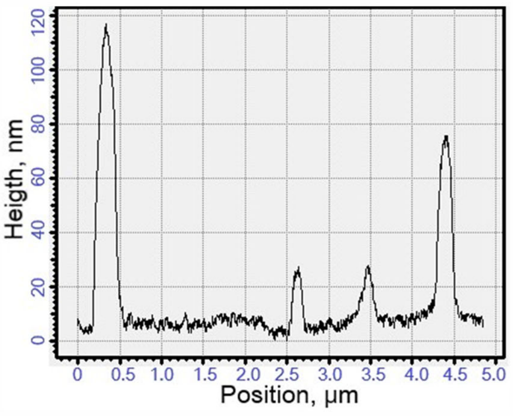

(**b**)

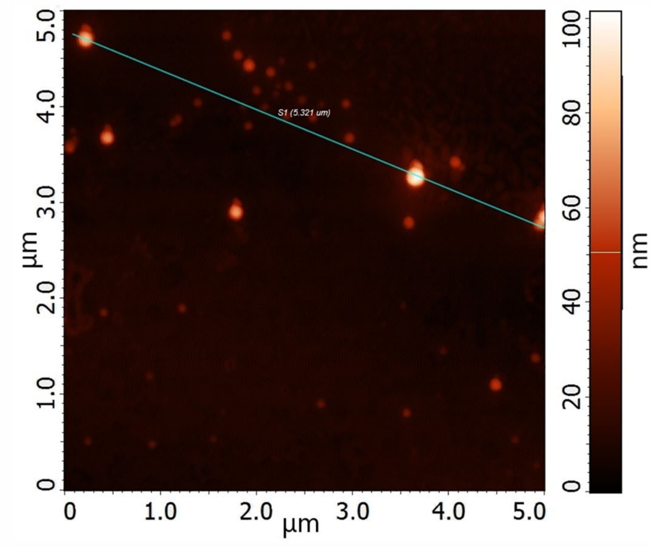

(**c**)

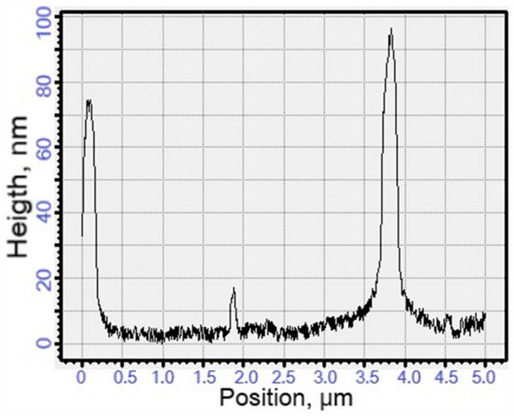

(**d**)

Figure S6. Fluorescence images of K562 cells after 2 h incubation with Dox-containing formulations. C_{Dox} = 2 μM. Dox channel: λ_{exci} = 500–560 nm, λ_{exci} = 590–700 nm. The scale segment is 100 μm. DoxM1 – Dox in Chit5-LA.

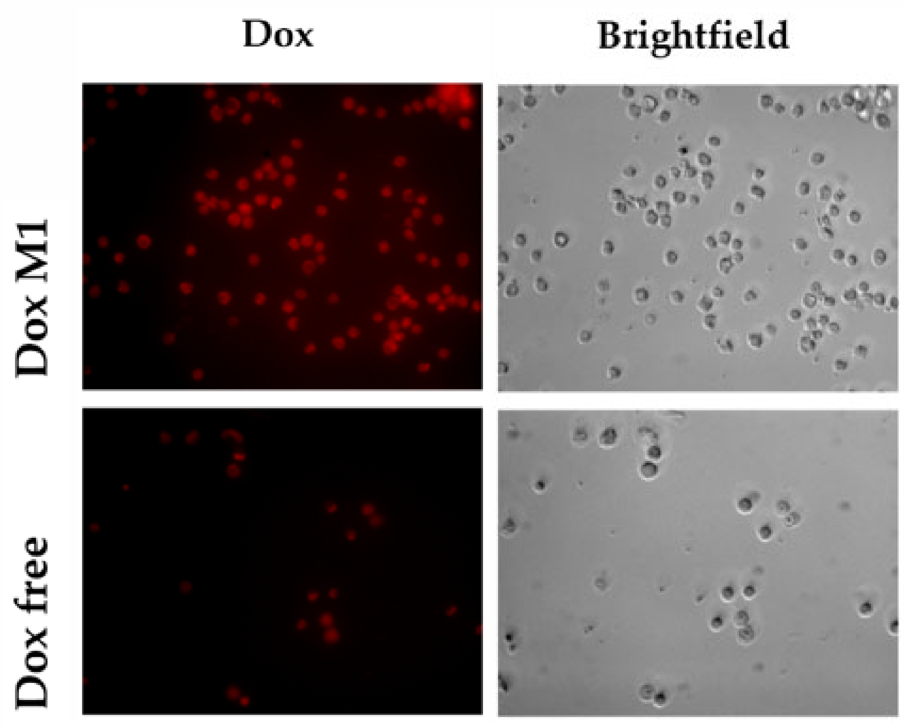

Supplement: Supplementary file 1 [file polymers-16-02132-s001.zip › polymers-3066319-supplementary.pdf]
